# Prevalence of Human Norovirus GII.4 Sydney 2012 [P31] between 2019 and 2021 among Young Children from Rural Communities in South Africa

**DOI:** 10.3390/v15081682

**Published:** 2023-08-02

**Authors:** Ronewa Khumela, Jean-Pierre Kabue, Marcia Terezinha Baroni de Moraes, Afsatou Ndama Traore, Natasha Potgieter

**Affiliations:** 1 Department of Biochemistry and Microbiology, Faculty of Science, Engineering and Agriculture, University of Venda, Private Bag X5050, Thohoyandou 0950, South Africa; kabuejeanpierre@yahoo.fr (J.-P.K.); afsatou.traore@univen.ac.za (A.N.T.); natasha.potgieter@univen.ac.za (N.P.); 2 Laboratory of Comparative and Environmental Virology, Oswaldo Cruz Institute, Oswaldo Cruz Foundation (FIOCRUZ), Avenida Brazil, 4365-Manguinhos, Rio de Janeiro 21040-360, RJ, Brazil; marciaterezinha4@gmail.com

**Keywords:** norovirus, acute gastroenteritis (AGE), symptomatic, asymptomatic, GII and GI genogroups, GII.4 Sydney 2012 [P31], GII4 Sydney 2012

## Abstract

Acute gastroenteritis (AGE) accounts for considerable morbidity and mortality in the paediatric population worldwide, especially in low-income countries. Human norovirus (HNoV), particularly GII.4 strains, are important agents of AGE. This study aimed to detect and characterise HNoV in children with and without AGE. Between 2019 and 2021, 300 stool samples (200 AGE and 100 without AGE) were collected from children below 5 years of age referred to the healthcare facilities of the rural communities of Vhembe District, South Africa. After detection using real-time RT-PCR, HNoV positive samples were subjected to RT-PCR and Sanger sequencing. Partial nucleotide sequences (capsid/RdRp) were aligned using the Muscle tool, and phylogenetic analysis was performed using MEGA 11. The nucleotides’ percent identity among HNoV strains was compared using ClustalW software. A significant difference in HNoV prevalence between AGE children (37%; 74/200) and non-AGE (14%; 14/100) was confirmed (*p* < 0.0001). Genogroup II (GII) HNoV was predominant in AGE children (80%; 59/74), whereas most non-AGE children were infected by the GI norovirus genogroup (64%; 9/14). GII.4 Sydney 2012 [P31] strains were dominant (59%; 19/32) during the study period. A phylogenetic analysis revealed a close relationship between the HNoV strains identified in this study and those circulating worldwide; however, ClustalW showed less than 50% nucleotide similarity between strains from this study and those from previously reported norovirus studies in the same region. Our findings indicate significant changes over time in the circulation of HNoV strains, as well as the association between high HNoV prevalence and AGE symptoms within the study area. The monitoring of HuNoV epidemiology, along with stringent preventive measures to mitigate the viral spread and the burden of AGE, are warranted.

## 1. Introduction

Viral acute gastroenteritis (VAGE) is a common disease in both high- and low-income countries [1]. Since its discovery, human norovirus (HNoV) has emerged and become the common cause of VAGE worldwide, accounting for over 700 million cases and 219,000 deaths annually [2,3,4]. Although norovirus affects people of all ages, it remains a significant cause of paediatric morbidity and mortality [1], especially in low-income countries [5,6]. The global prevalence of norovirus ranges from 12% to 20.2% [7,8,9]; however, a higher prevalence has also been reported in Africa [10,11]. The spread of norovirus is mainly facilitated by the faecal oral route and poor hygiene practice [12].

Norovirus is a genus in the *Caliciviridae* family composed of enveloped, positive sense, genetically diverse viruses with an approximately 7.5 kb single-stranded RNA genome [13,14]. The norovirus genome consists of three ORFs (open reading frames), including ORF1, which encodes a large polyprotein that is cleaved by viral protease to generate nonstructural proteins (NS1/2-7); ORF2, which encodes a major capsid protein VP1; and ORF3, which encodes a minor structural protein VP2 [13,15]. The capsid protein consists of the shell (S) domain and two protruding (P) subdomains. Norovirus vaccine candidates currently in trial are VP1-based [16]. Amongst the 10 genogroups (GI-GX) of norovirus, VAGE is mainly caused by GI, GII, and GIV, with GII being the most predominant genogroup worldwide. Based on VP1 amino acid sequences and partial RdRp nucleotide sequences, norovirus genogroups are subdivided into 48 capsid genotypes and 60 P-types [17]. GII.4 is the most common genotype infecting humans worldwide and has been implicated in serious pandemics for about three decades [18]. The majority of HNoV-related deaths are predominantly reported in low-income countries [5,6], and over the years, HNoV infections increasingly impose an enormous economic burden in developed countries [19].

The predominance of GII.4 Sydney 2012 [P31] strains has been increasingly reported [20,21,22]. The capacity of a viral strain or variant to remain dominant in a given geographical area is defined as epidemiological fitness [23,24,25]. The features involved can include viral and host characteristics, though they cannot be easily quantified [23]. Observational data, such as changes in the prevalence, distribution, and composition of viral genotypes, may be used to quantify the epidemiological fitness of a virus [24].

In South Africa, the GII.4 Sydney 2012 [P31] strain was detected in children hospitalised with AGE from an urban area [26]. Kabue et al. [27] worked on outpatients from a rural area in South Africa, who also showed a high prevalence of GII.4 norovirus strains. There is poor WASH (water, sanitation, and hygiene) practice in rural communities [28], which may aid the spread of pathogens. Understanding the epidemiological fitness of prevalent viral genotypes is critical for therapeutic interventions and preventive strategies to minimise the spread. Therefore, continuous surveillance is critical in this era of vaccine development to provide up-to-date genetic information and understanding of norovirus epidemiology in areas with a history of high norovirus prevalence where preventive measures would have an enormous impact [16]. This study aimed to give an update on norovirus prevalence and genetic characteristics in children within the rural communities of Vhembe District in South Africa.

## 2. Materials and Methods

### 2.1. Ethics

The study protocol and consent procedures were approved by the ethics committees of the Department of Health, Limpopo Province (LP-2018-07-016) and the Research Directorate of the University of Venda (Ref. SMNS/18/MBY/07/2505). Written, informed consents were given by the parents or children’s guardians before sample collection.

### 2.2. Study Population and Sample Collection

Between 2019 and 2021, a cross-sectional study on children under five years of age living in the rural communities of Vhembe District, Limpopo, South Africa, was performed. The study population was subdivided into 2 groups, symptomatic (children with AGE symptoms) and asymptomatic (children without any AGE symptoms for at least one month) [12]. Inclusion for AGE children was based on the passing of at least 3 or more loose stools within 24 h [29]. Nonbloody diarrhoeal stool samples (*n* = 200) were randomly collected at different primary healthcare clinics and hospitals within the district. For healthy controls, 100 stools samples were collected in healthcare facilities within the same region. Information relating to the personal details, clinical symptoms, and living conditions (e.g., water source, presence of livestock) of the patients was obtained on preprinted investigation forms. In hospitalised cases, only children admitted within 24 h were considered for this investigation. All specimens were transported to the University of Venda Microbiology Laboratory and stored at −20 °C for further analysis.

### 2.3. RNA Extraction and Human Norovirus Detection

Viral nucleic acid was extracted using Boom method [30] from pea-sized stools suspended in 500 μL of phosphate buffer saline (PBS). This method is based on the lysis and nuclease-inactivating properties of the chaotropic agent guanidium thiocyanate, together with the nucleic acid-binding properties of silica particles. RNA extracts were stored at −20 °C prior to norovirus detection. RIDAGENE real-time RT-PCR (r-Biopharm AG, Darmstadt, Germany) kit’s viral stool panel (PG1415) was used to detect and differentiate norovirus genogroup I and II. This assay, according to the manufacturers, is thought to not cross-react with other common enteric pathogens. The assay has 98% sensitivity and specificity [31] and includes internal control to monitor the extraction efficiency and amplification inhibition. The platform used for qPCR was Rotor-gene Corbett 6000. Norovirus positivity threshold cycle value was considered up to ≤37 (Ct) for GII and ≤35 for GI, with display of sigmoid curve [32,33].

### 2.4. Norovirus RT-PCR and Genotyping, ClustalW Alignment, and Phylogenetic Analysis

Norovirus positive RNA extracts were amplified with genogroup-specific polymerase–capsid (PC) assay [32] using Qiagen OneStep Reverse Transcription-PCR kit (Qiagen, Germantown, MD, USA) for the purpose of nucleotide sequencing. Previously described primers [34,35] for GI MON432/GISKR (579 bp) were used for this assay under the following PCR conditions: reverse transcription at 42 °C for 30 min, activation of Taq polymerase at 95 °C for 15 min, and 40 cycles of PCR amplification at 95 °C, 50 °C, and 72 °C for 1 min each, followed by 10 min at 72 °C and cooling down to 4 °C. MON431/GIISKR (570 bp) for GII was used with one adjustment on the PCR conditions annealing at 56 °C. Norovirus positive extracts that could not be amplified using previous conditions were subjected to another round of amplification (seminested PCR) by the primers COG2F/G2SKR(GII) and COG1F/G1SKR (GI), which generate 390 pb and 380 pb fragments, respectively [34,36]. The amplicons that still could not generate a band were subjected to OneStep Ahead RT-PCR using GISKR/F and GIISKR/F primers that produce 330 bp and 344 bp, respectively [34]. The norovirus amplicons were separated using electrophoresis on 2% agarose gel stained with ethidium bromide and specific-sized band visualised using UV light transilluminator. All amplicons visualised at the expected size were sent for partial Sanger sequencing using 3730XL Genetic Analyzer POP7^TM^ (Thermo-Scientific, Waltham, MA, USA) at Inqaba Biotec^TM^ (Pretoria, South Africa). The same primers for conventional PCR were used during Sanger sequencing. Raw sequences were read and edited via Finch TV (version 1.4) (Geospiza, Seattle, WA, USA). The edited nucleotide sequences were then compared with reference strain accessed on NCBI blast tool (accessed on 13 March 2023) at http://www.ncbi.nlm.nih.gov/. Edited sequences were genotyped using the online noronet platform at http://www.rivm.nlm/norovirus/typingtool (accessed on 15 March 2023) and human calicivirus typing tool HuCaT (Atlanta, GA, USA) at https://norovirus.ng.philab.cdc.gov/ (accessed on 15 March 2023). The norovirus genotypes obtained in this study were submitted to NCBI GenBank and have been assigned the following accession numbers: OM948743-OM948745, OM961396-OM961399, OM970798-OM970802, OM985015-OM985018, OM993270-OM993272, ON005452, ON008179, OP257195-OP257196, OP600464-OP600468, and OQ048857-OQ048862.

The ClustalW alignment tool was used to compare aligned sequences’ nucleotide percent identity between current genotyped norovirus (*n* = 34) and previously published norovirus sequences (*n* = 14) in the same study area [37]. After sequence input, ClustalW is able to show nucleotide similarities in percentages amongst all sequences.

Phylogenetic analysis was performed to investigate the genetic relationship between norovirus strains detected in this study and others circulating worldwide. The reference strains from GenBank with best hit of >80% similarity to the study’s query sequences were randomly selected for phylogenetic analysis using MEGA 11 [38]. Additional strains were selected based on location to fully investigate the relatedness of worldwide circulating strains over time.

### 2.5. Statistical Analysis

Data were captured on Excel spreadsheet. Statistical significance was determined by calculating *p*-values using Chi-square and Fisher’s exact test on GraphPad Prism 9 (GraphPad Inc., San Diego, CA, USA).

## 3. Results

### 3.1. Human Norovirus Prevalence and Sample Characteristics

This study presented the prevalence of HNoV between 2019 and 2021 (Table 1) within the rural communities of Vhembe District. The prevalence of HNoV was 37% (74/200) in symptomatic and 14% (14/100) in asymptomatic individuals. The difference in symptomatic and asymptomatic HNoV prevalence was proven to be statistically significant (*p* < 0.0001). HNoV significantly (*p* = 0.0125) affected more hospitalised patients (60%; 44/74) than outpatients (41%; 30/74) during the investigation. The majority of study participants were males (58%) compared to females (42%), and a higher HNoV prevalence was recorded in males (66%) than females (34%). Children aged 6–23 months (65%) were predominantly infected by HNoV, followed by children aged 0–5 months (20%).

In addition, HNoV infections were mainly found in diarrhoea cases associated with other related symptoms (89%) than diarrhoea alone (11%) amongst the study participants. The most common norovirus infection symptoms included dehydration (82%), vomiting (73%), and fever (49%), as shown in Table 1. There was an increase in HNoV in children with dehydrating diarrhoea from 2019 (27%; 10/37) to 2020 (45%; 30/66) and 2021 (92%; 89/97). Children with watery diarrhoea had high infection (62%) compared to those releasing formed stools (38%). The majority of HNoV-positive (82%) children reported having diarrhoea that lasted no more than 3 days.

Children who reported coming from households using treated tap water showed a higher prevalence of HNoV (65%; 71%) compared to those who depend on untreated river water (15%; 29%) or mixed water sources (20%) in both the symptomatic and asymptomatic groups, respectively. There was a slight difference in HNoV positivity amongst participants who use pit toilets (55%) and flush toilets (45%). There was no statistical significance in HNoV-positive breastfed children with AGE (69%) compared to nonbreastfed children (31%) (*p* = 0.3732). Similarly, HNoV was detected in the majority of asymptomatic children who were breastfed (71%) compared to those not breastfed (29%) (*p* = 0.4812). The presence or absence of livestock data, as provided by participants, was inconsistent; however, people who lived with animals had a high HNoV positivity rate (39%; 21%) compared to those without (12%; 7%) in both the symptomatic and asymptomatic groups.

#### 3.1.1. HNoV Genogroup and Genotype Distribution

We observed an increase in distribution of GII over the years from 2019 (3/59) to 2020 (15/59) and 2021 (41/59) in children with AGE. GII infections predominated (80%; 59/74) GI (12%; 9/74) in children with AGE. Children without AGE were infected with GI HNoV (64%; 9/14) more than GII (36%; 5/14) (Table 2). The predominance of GII was most common in hospitalised patients (86%) than in outpatients (70%). GI/GII coinfection was less common in this study. The median of GII Ct values between the symptomatic (32.25) and asymptomatic (35.02) groups was slightly different. Similar findings were observed in the mean GI Ct values in the symptomatic (32.16) and asymptomatic (32.50) groups.

Out of 59 cases of HNoV GII detected in symptomatic children, 54% (32/59) were successfully amplified and genotyped. In asymptomatic participants, 40% (2/5) of GII strains were genotyped. Only GII.4 Sydney 2012 [P31] recombinants and the capsid genotype GII.4 Sydney 2012 were successfully amplified and sequenced in this study. An analysis of the junction region of ORF1/2 revealed the predominance of GII.4 Sydney 2012 [P31] recombinants in symptomatic (59%; 19/32) and asymptomatic HNoV (40%; 2/5) infections. The GII.4 Sydney 2012 [P31] genotype was more common in hospitalised children (74%; 14/19) compared to outpatients (26%; 5/19). The distribution of the GII.4 Sydney 2012 genotype in inpatients and outpatients was similar. The remaining positive amplicons (41%; 13/32) could only be typed on the capsid fragment as GII.4 Sydney 2012 genotypes. The HNoV GI group and other GII positive samples could not be typed, possibly due to low viral load and the impact of electricity load shedding on our storage facilities.

#### 3.1.2. Comparison of Nucleotide Sequences among HNoV Strains Circulating in Vhembe District, South Africa

The ClustalW percent identity matrix (Data S1) between norovirus strains previously reported in the study area [37] and those characterised from the current survey showed a significantly low similarity of less than 50% in nucleotide sequences. The change in nucleotide sequences was observed in a period of about 4 to 5 years. When comparing HNoV sequences obtained only from this investigation, we noted that they were all similar, with a nucleotide percent identity of more than 90%.

### 3.2. Phylogenetic Analysis

**Figure 1:**
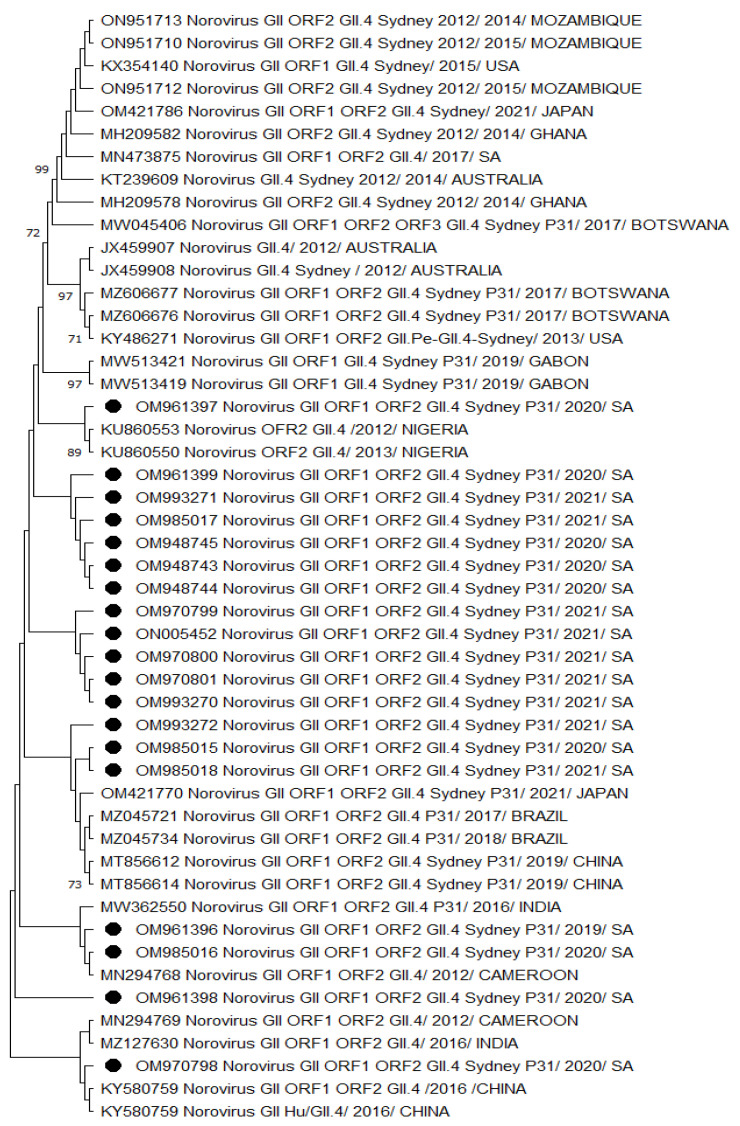
Phylogenetic analysis of the dual typed (polymerase and capsid junction region, 570 bp (with MON431/G2SKR)) HNoV nucleotide sequence circulating in Vhembe District (South Africa) in 2019–2021. Phylogenetic tree was set using neighbour joining method. Round black dots indicate HNoV genotyped in this study, and reference sequences were randomly selected from GenBank, with their respective accession numbers based on high similarity with our study sequences. All positions containing gaps and missing data were eliminated. The evolutionary distances were computed using the *p*-distance method and are in the units of the number of base differences per site. Evolutionary analyses were conducted in MEGA 11 (10.0.5) and bootstrap tests (1000 replicates) based on the Kimura two-parameter model. Only bootstrap values greater than 70% are shown.

**Figure 2:**
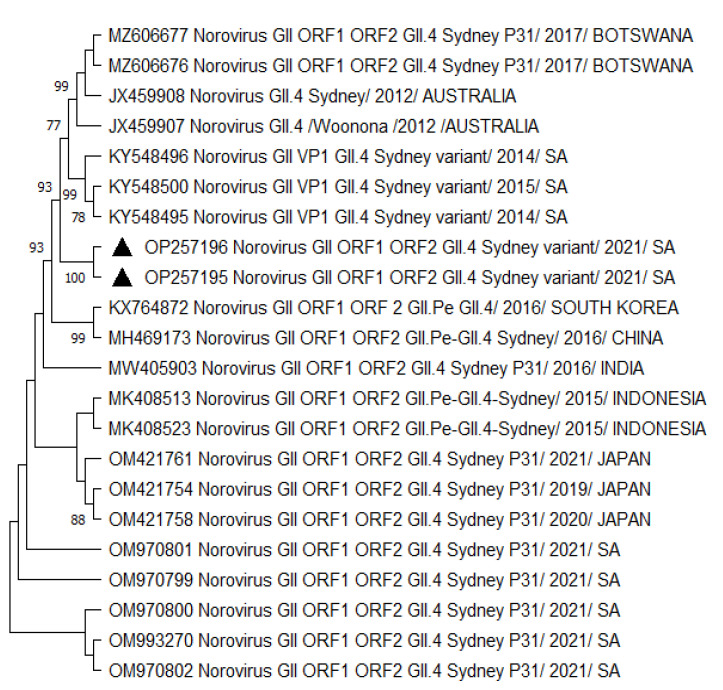
Phylogenetic tree based on 380 bp (with COG2F/G2SKR) capsid/RdRp junction region of HNoV nucleotide sequence circulating in Vhembe District (South Africa) in 2019–2021. Neighbour joining method was used in building the tree. Triangle black dots indicate the HNoV genotypes obtained during this study, and reference sequences were randomly selected from GenBank, with their respective accession numbers based on high similarity with our study sequences. All positions containing gaps and missing data were eliminated. The evolutionary distances were computed using the *p*-distance method and are in the units of the number of base differences per site. Evolutionary analyses were conducted in MEGA 11 (10.0.5) and bootstrap tests (1000 replicates) based on the Kimura two-parameter model. Bootstrap values higher than 70% are shown.

A phylogenetic analysis of HNoV GII.4 Sydney 2012 [P31] sequences from Vhembe showed multiple clades (Figure 1), clustering mostly among each other, indicating a close relatedness of the circulating HNoV strains in the area. Amongst these clusters, we observed HNoV strains from India, Cameroon, and China sharing a common ancestor; however, Australian strains from 2012 were in a very distant branch, indicating the existence of a considerable difference with those that have been circulating recently. The nucleotide sequence identity of norovirus strains obtained from this study showed 95% similarities. Figure 2 showed that the most closely related strains were those from China and previously characterised South African strains.

**Figure 3:**
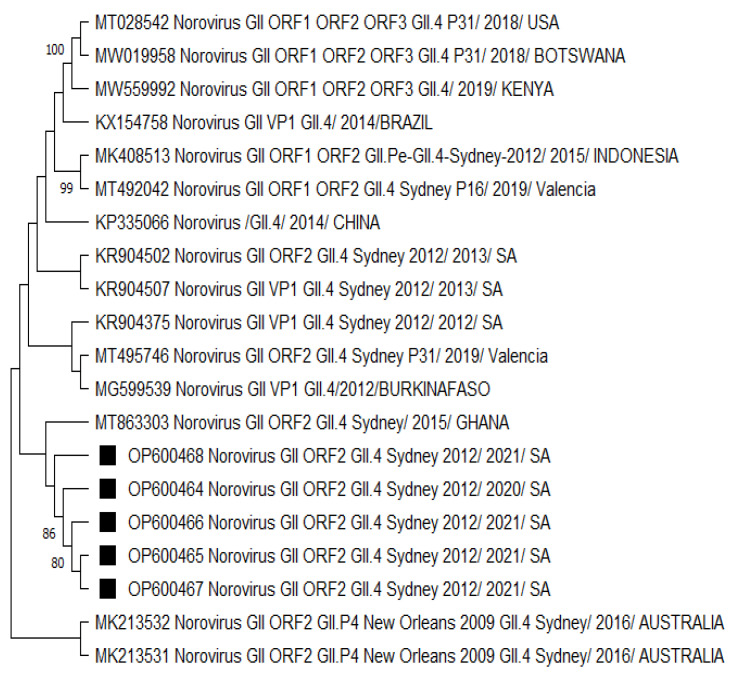
Phylogenetic analysis of the partial typed 340 bp (with G2SKF/R) capsid region of HNoV nucleotide sequence circulating in Vhembe District (South Africa) in 2019–2021. Phylogenetic tree was set using neighbour joining method. Squared black dots indicate HNoV genotyped in this study, and reference sequences were randomly selected from GenBank, with their respective accession numbers based on high similarity with our study sequences. All positions containing gaps and missing data were eliminated. The evolutionary distances were computed using the *p*-distance method and are in the units of the number of base differences per site. Evolutionary analyses were conducted in MEGA 11 (10.0.5) and bootstrap tests (1000 replicates) based on the Kimura two-parameter model. Only bootstrap values greater than 70% are shown.

The GII.4 Sydney 2012 strains analysed in Figure 3 were mostly related to a norovirus strain from Ghana. The neighbouring clades consisted of norovirus strains from Burkina Faso and the other previously listed countries in Figure 1 and Figure 2. There was no significant difference in the capsid- and polymerase-protein-based phylogenetic analysis (Figures S2 and S3) compared to the nucleotide-based trees. However, the capsid-protein-based tree confirmed that strains from neighbouring countries (Botswana and Mozambique) shared a common ancestor with the HNoV strains detected in this study (Figures S1 and S4), reflecting the role of border proximity.

## 4. Discussion

Human norovirus is a global public health problem associated with diarrhoea disease worldwide. In this study, we attempted to understand the epidemiological fitness of norovirus strains by analysing the change in prevalence, distribution, and composition of norovirus genotypes. A high prevalence of HNoV was previously reported in the rural communities of Venda, South Africa [27]. Table 2 shows that HNoV strains were still circulating at a high rate (37%) within the study area. This rate is higher than the pooled prevalence of norovirus strains in Africa [7] but comparable to what has been reported, particularly in Ghana [10]. Norovirus has now emerged as the leading cause of AGE worldwide [3], especially in regions where the rotavirus vaccine has been introduced [39]. This study was conducted in the rural communities of Vhembe District, South Africa, where rotavirus vaccines have been introduced since 2009.

There was a significant difference (*p* < 0.0001) in the HNoV infection rate between the symptomatic (37%) and asymptomatic (14%) groups. This observation indicated the considerable role of norovirus in the symptomatic presentations of AGE in Vhembe District. A previous norovirus survey in this study area did not find a statistical difference between symptomatic and asymptomatic subgroups [27]. The findings suggest that norovirus is strongly associated with VAGE in this region, as reported in other low- and middle-income countries [40,41]. HNoV infections level were higher (59%) in hospitalised patients compared to outpatients (41%), suggesting that norovirus is actively involved in AGE symptoms and diarrhoea disease (*p* = 0.0129). In a recent South African study within urban areas, norovirus was associated with severe diarrhoea in hospitalised children [26]. Norovirus severity and complications have been previously described elsewhere in countries like Qatar and Taiwan, respectively [42,43]. However, our data did not include other gastroenteritis-causing pathogens.

In this study, HNoV predominated among children between 6 and 23 months old. Recently published data showed that norovirus disease burden is mostly experienced at this age range in African countries [40]. A similar finding was observed in the MALED group study, wherein norovirus infection peaked after 6 months [44]. This is a critical age for individual and immune system development with increased exposure. Available data showed high vulnerability to infection in children, especially those under 2 years [10,44]. At this age, children are thought to have lost maternal antibody protection [45] and are more engaged in increased physical activities, which could lead to more environmental exposure. The results in this study support other findings which suggested that vaccine development should prioritise and target the low age range around six months and above. Approximately 85% of children under 2 years could be protected with this implementation [10,44,45].

Although the breastfeeding of children has been associated with protection against infections [46,47,48], no statistically significant protection against HNoV infection was seen from the present study (*p* = 0.3732). The results agree with recent findings [49,50]. In the first six months of life, the WHO and UNICEF still recommend exclusive breastfeeding with its association to AGE protection because an infant’s immunity is boosted by maternal antibodies through breastfeeding [51]. Furthermore, Ghosh et al. [52] recently demonstrated the transmission of enteric viruses from infants’ salivary glands to mothers’ mammary glands during suckling, leading to an increased flow of maternal milk secretory IgA antibodies.

Male children were more infected than females during this investigation. Previous studies indicated that prevalence of viral infections is typically higher in males than females [21,53]. Jaillon et al. [53] reported that females are thought to have stronger immunity compared to males, as some genes that aid during immune response are located in the X chromosome.

Although only the clinical samples were tested, the overall living conditions data provided were not statistically significant. However, the presence of HNoV in both pit/flush toilets, treated water, and in children living with domestic animals suggests poor hygiene practices in the study area. Previous data in the study area have shown faecal contamination of multiple water sources, including tap and household storage containers, with implications in distribution networks and unhygienic practices [54]. Furthermore, Ayukekbong et al. [55] reported norovirus presence in tap water in Cameroon, implying possible resistance to chlorine used in the treatment of water, as previously demonstrated [56].

Due to COVID-19 pandemic lockdown restrictions, no seasonal distribution data could be provided during the investigation. However, throughout the study period, HNoV infections showed a yearly increase, with the highest number recorded in 2021. This observation was consistent with the prediction of the rise in norovirus cases upon the relaxation of COVID-19 regulations, as demonstrated by O’Reilly et al. [57]. Data indicating both the rise and decline in norovirus cases during the COVID-19 pandemic were reported [58,59]. Furthermore, norovirus outbreaks occurred during the COVID-19 pandemic in China and Japan [22,60].

HNoV GII infections predominated in symptomatic cases, especially hospitalised children, supporting the view that they should be prioritised during vaccine development. Our findings are comparable with studies demonstrating the role of GII in symptomatic infections [42,61,62]. Genogroup II strains have been associated with the majority of outbreaks worldwide [15,63]. In contrast, the GI group was found to be more prevalent amongst control cases in this study. These findings agree with the other studies outcomes suggesting that GI viruses are usually associated with mild infection, while GII viruses are mostly associated with disease symptoms [27,64].

Although the outcome of norovirus epidemiology studies may vary in each region, both GI and GII are epidemiologically important groups requiring continuous surveillance. There was no significant difference between the norovirus GI viral load in symptomatic and asymptomatic norovirus-positive samples. Similarly, in GII infections, the difference in NoV Ct values was insignificant, although a slightly higher median was observed in the asymptomatic group compared to the symptomatic group. Contrary to these findings, a previous investigation in the study area found a significant difference in Ct values between GI and GII groups [27].

The GII.4 Sydney 2012 [P31] variant predominated in this study, particularly among symptomatic inpatients. The GII.4 Sydney 2012 strain was first detected in Australia and later spread throughout the world, with new variants emerging almost every two to three years [65]. The majority of HNoV outbreaks are caused by GII.4 genotypes, which have led multiple pandemics since the mid-1990s [66]. In 2000 and 2004, US95/96 was replaced by two new GII.4 variants, Farmington Hills and Hunter GII.4 variants [67,68]. Other strains were also found in 2006 and 2009 [69]. The GII.4 Sydney variant dominates in most of countries, including South Africa. GII.4 strains have been associated with severe outcomes of norovirus infection and less asymptomatic infection [65,70]. Similarly, our study findings indicate the increase in GII norovirus strains in dehydrating diarrhoea from 2019 to 2020 and 2021.

Since 2012 to date, the Sydney variant, particularly the GII.4 Sydney 2012 [P31] strain, became epidemiologically dominant and persistent through mutations and recombination [65]. The ability of GII.4 variants to evolve at a faster rate on capsid accounts for their epidemiological fitness [71]. Further analysis of GII.4 Sydney 2012 [P31] nucleotide sequences in this study showed evidence of considerable change and possible mutations in strains over time. We observed less than 50% similarity in the nucleotide sequences of HNoV GII.4 strains obtained in this study compared to previously published data (Data S1). The genetic variation of HNoV strains over the years ascertains how HNoV may continue to affect vulnerable communities in different countries, with the possibility of the emergence of new variants in the near future [22,60]. The variation of sequence nucleotides could explain the viral adaptability and epidemiological fitness or dominance in the area [72]. The molecular characteristics of GII.4 Sydney [P31] strains during an outbreak in China suggested that the strains were undergoing evolution, which could lead to the emergence of new variants [22].

It is unclear what may have selectively facilitated the epidemiological fitness and spread of the HNoV GII.4 Sydney 2012 P31 strain amid COVID-19 in the study area. However, the same strain was reported to be circulating in Japan and China during COVID-19 [22,60]. The features of epidemiological fitness are not easily quantifiable [24]. More data, including a vast category of viral, host, and environmental characteristics, are needed to understand the dominance of the norovirus GII.4 Sydney 2012 [P31] strain. Recent data reported the predominance of the GII.4 Sydney 2012 [P31] strain in norovirus-associated diarrhoea [20,21], suggesting that this genotype may remain dominant for a while due to its fitness capacity in different populations. Other genotypes were not characterised in this study; this could be due to the devastating impact of the South African national electricity load shedding schedule on laboratory cold storage units, which may have selectively favoured the survival of GII.4 Sydney 2012 [P31] strains. Furthermore, the majority of samples were amplified using one-step RT-PCR for the purpose of dual typing, which may result in a high error rate during amplification compared to high-fidelity polymerases [73]. This could have limited our typing.

A phylogenetic analysis revealed the close relatedness among norovirus GII.4 Sydney 2012 [P31] recombinants (Figure 1) circulating within the study region. A similar observation was made for GII.4 Sydney 2012 strains (Figure 2 and Figure 3), which were all phylogenetically related to each other. We noted the GII.4 Sydney 2012 [P31] strains were closely related to the NoV strains circulating in Nigeria, Gabon, Cameroon, China, and Japan. Norovirus GII.4 Sydney 2012 [P31] from neighbouring countries such as Mozambique and Botswana shared a common ancestor (Figures S1 and S4). The circulation of closely related strains in different countries, especially those within the same continent, may be attributed to border proximity. However, in some cases, norovirus strains were closely related to other circulating strains outside of Africa, which is thought to have been due to population movement prior to the COVID-19 pandemic lockdown. Sequence analysis showed that GII.4 Sydney 2012 strains (Figure 2 and Figure 3) are most phylogenetically related to the HNoV strains recently reported in Japan [60] in sporadic cases and outbreaks and elsewhere in countries such as China [74], Botswana [75], and Ghana [10]. The GII.4 variants became predominant in outbreaks starting from the early 1990s until the current circulating GII.4 Sydney 2012.

Although the GII.4 Sydney strains shared a common ancestor, the data from the present study suggested that there has been a change over the years in the predominant GII.4 Sydney 2012 strains first detected in Australia (2012) compared to the recent circulating variants (Figure 1, Figure 2 and Figure 3) by distant clusters. This phenomenon is confirmed by the nucleotide sequence variations observed in the present investigation between different norovirus strains over the years.

## 5. Conclusions

HNoV continues to be responsible for VAGE outbreaks affecting various populations worldwide. This study provides valuable insight into the dominance of the GII.4 Sydney 2012 [P31] genotype and its genetic variation over time. More efforts are needed in the control of norovirus circulation and spread, especially in highly affected rural communities with inadequate WASH conditions. It is imperative to monitor the genetic changes and epidemiology fitness of HNoV strains and the possible emergence of new variants in this era of norovirus vaccine development.

## Figures and Tables

**Table 1:** Demographic characteristics, clinical features, and HNoV prevalence among children with AGE from Vhembe District, South Africa, in 2019–2021.

	Symptomatic		Asymptomatic	
	Total (%)	HNoV+ (%)	Total (%)	HNoV+ (%)
Detection rate (%)	*n* = 200	*n* = 74 (37)	*n* = 100	*n* = 14 (14)
Setting				
Outpatients	104 (52)	30 (41)	100 (100)	14 (14)
Hospitalised	96 (48)	44 (59)	0	0
Gender				
Males	116 (58)	49 (66)	53 (53)	8 (57)
Females	84 (42)	25 (34)	47 (47)	6 (43)
Age in months				
0–5	49 (25)	15 (20)	21 (21)	2 (14)
6–23	124 (62)	48 (65)	59 (59)	10 (71)
24–60	27 (14)	11 (15)	20 (20)	2 (14)
Symptoms				
Diarrhoea only	48 (24)	8 (11)		
Diarrhoea and other symptoms	152 (76)	66 (89)		
Other symptoms seen together with diarrhoea				
Dehydration	132 (66)	61 (82)		
Vomiting	119 (60)	54 (73)		
Fever	79 (40)	36 (49)		
Abdominal pain	29 (15)	15 (20)		
Stool appearance				
Watery	103 (52)	46 (62)		
Formed	97 (49)	28 (38)	100 (100)	14 (14)
Duration of diarrhoea				
3 days	163 (82)	62 (84)		
>3 days	36 (18)	12 (16)		
Interval				
1–3 days	161 (81)	61 (82)		
3 days +	39 (20)	13 (18)		
Living conditions				
Treated water	131 (66)	48 (65)	73 (73)	10 (71)
Untreated water	37 (19)	11 (15)	13 (13)	4 (29)
Mixed	32 (16)	15 (20)	14 (14)	
Pit toilets	111 (56)	41 (55)		
Flush toilets	89 (46)	33 (45)		
Breastfeeding	130 (65)	51 (69)	63 (63)	10 (71)
Not breastfed	70 (35)	23 (31)	37 (37)	4 (29)
Livestock	67 (34)	29 (39)	32 (32)	3 (21)
No Livestock	21 (11)	9 (12)	7 (7)	1 (7)

**Table 2:** Distribution of HNoV genogroup and genotype in children from Vhembe District, South Africa.

	Symptomatic			Asymptomatic
		Outpatients	Inpatients	
	*n* = 200 (%)	*n* = 104 (%)	*n* = 96 (%)	*n* = 100 (%)
Total HNoV+	74 (37)	30 (41)	44 (60)	14 (14)
Genogroups				
GI	9 (12)	4 (12)	5 (11)	9 (64)
GII	59 (80)	21 (70)	38 (86)	5 (36)
Mixed	5 (7)	2 (7)	3 (7)	
GII Genotypes	32			
GII.4 Sydney 2012 [P31]	19 (59)	5 (26)	14 (74)	2 (40)
GII.4 Sydney 2012	13 (41)	6 (46)	7 (54)	

## Data Availability

The data set, including parameters, considered during this investigation is available upon request to the corresponding author.

## Supplementary Materials

The following supporting information can be downloaded at: https://www.mdpi.com/article/10.3390/v15081682/s1, Data S1: Percent Identity Matrix created by Clustal2.1. Norovirus sequence numbers 1, 13–15, and 39–48 were obtained from previously conducted studies in Vhembe District. The rest of the sequences were produced during this investigation. Figures S1, S3 and S4: Capsid protein based phylogenetic tree of norovirus. Figure S2: RdRP proteins based phylogenetic tree of norovirus.
