# Supplementary material for: Prevalence of Human Norovirus GII.4 Sydney 2012 [P31] between 2019 and 2021 among Young Children from Rural Communities in South Africa
Source: Viruses. 2023 Aug 2;15(8):1682. doi: 10.3390/v15081682 (PMC10458076; doi:10.3390/v15081682)
Supplement: Supplementary file 1 [file viruses-15-01682-s001.zip › Figures S1–S4.pdf]

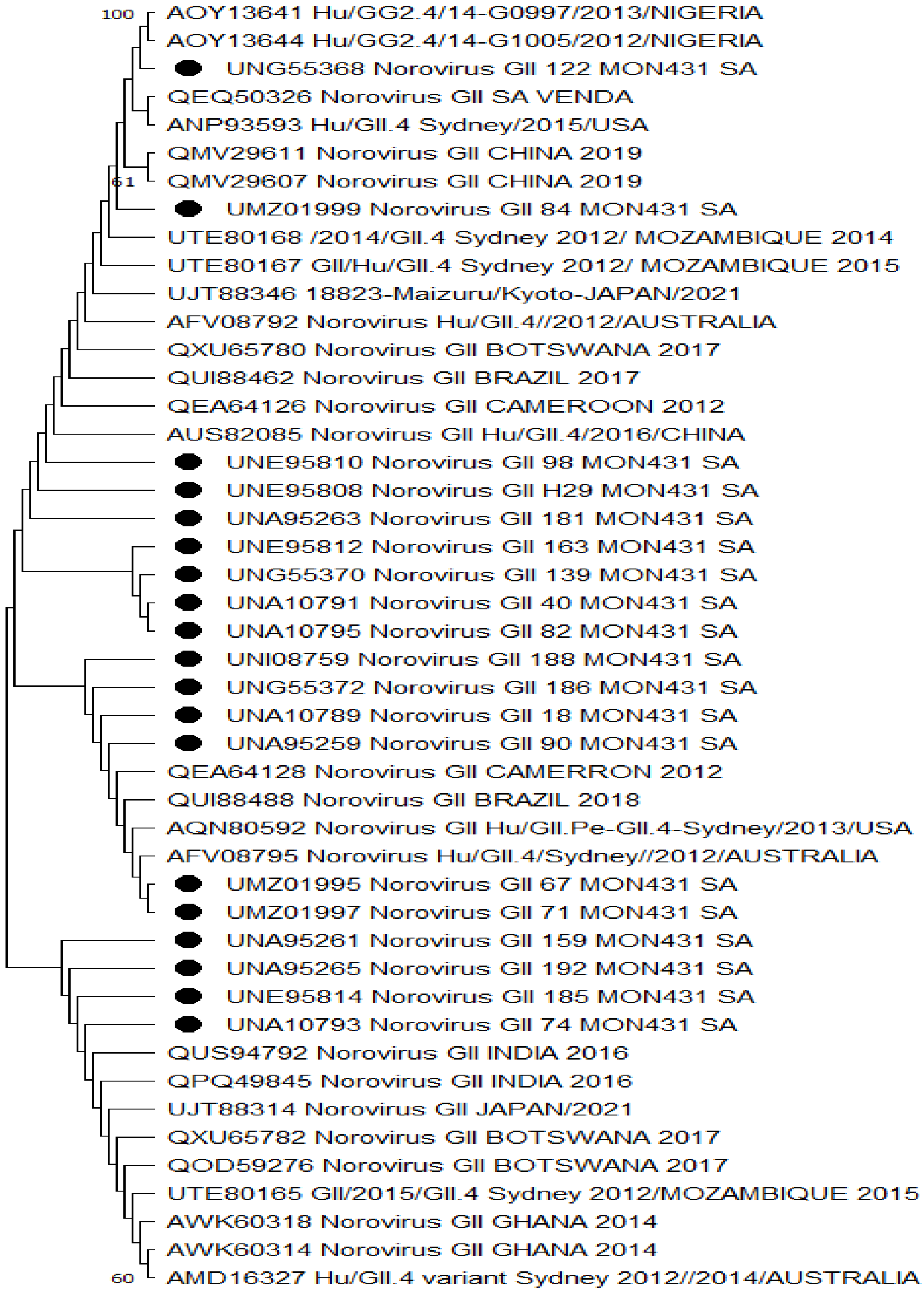

Figure S1. Phylogenetic analysis of capsid region proteins of HNoV GII.4 Sydney 2012 [P31] strains circulating in Vhembe district (South Africa) in 2019-2021. Phylogenetic tree was set using Neighbor-Joining method. Round black dots represent HNoV genotypes obtained during this investigation. Reference protein sequences were obtained from GenBank with their respective accession numbers based on nucleotide similarity with our study sequences when constructing a nucleotide based tree. All positions containing gaps and missing data were eliminated. The evolutionary distances were computed using the *p*-distance method and are in the units of the number of base differences per site. Evolutionary analyses were conducted in MEGA 11 (10.0.5) and bootstrap tests (1000 replicates) based on the Kimura two-parameter model. Only bootstrap values greater than 60% are shown.

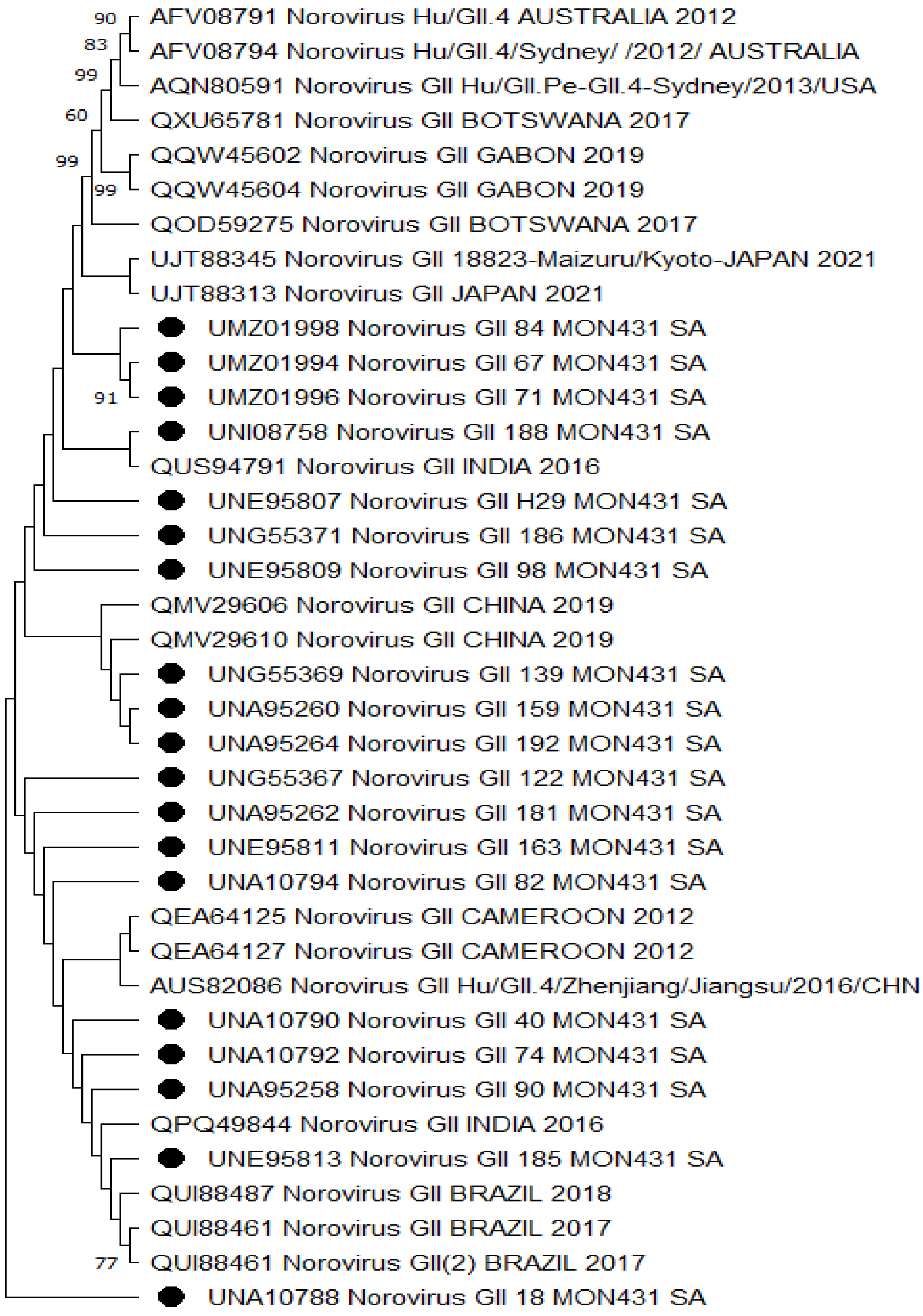

Figure S2. Phylogenetic analysis of polymerase region proteins of HNoV GII.4 Sydney 2012 [P31] strains circulating in Vhembe district (South Africa) in 2019-2021. Phylogenetic tree was set using Neighbor-Joining method. Round black dots represent HNoV genotypes obtained during this investigation. Reference protein sequences were obtained from GenBank with their respective accession numbers based on nucleotide similarity with our study sequences when constructing a nucleotide based tree. All positions containing gaps and missing data were eliminated. The evolutionary distances were computed using the *p*-distance method and are in the units of the number of base differences per site. Evolutionary analyses were conducted in MEGA 11 (10.0.5) and bootstrap tests (1000 replicates) based on the Kimura two-parameter model. Only bootstrap values greater than 60% are shown.

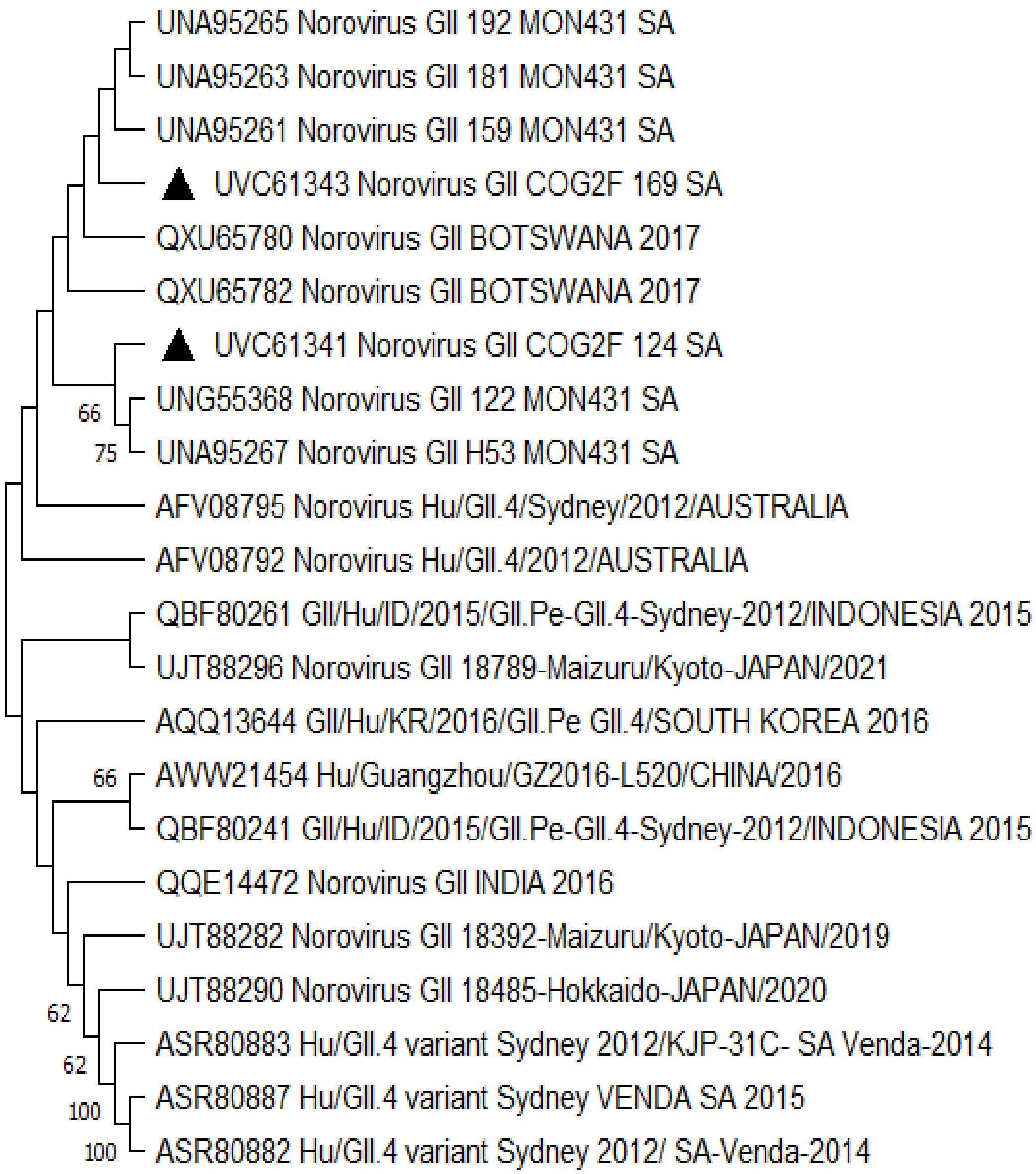

Figure S3. Phylogenetic analysis of capsid region proteins of HNoV GII.4 Sydney 2012 strains circulating in Vhembe district (South Africa) in 2019-2021. Phylogenetic tree was set using Neighbor-Joining method. Round black dots represent HNoV genotypes obtained during this investigation. Reference protein sequences were obtained from GenBank with their respective accession numbers based on nucleotide similarity with our study sequences when constructing a nucleotide based tree. All positions containing gaps and missing data were eliminated. The evolutionary distances were computed using the *p*-distance method and are in the units of the number of base differences per site. Evolutionary analyses were conducted in MEGA 11 (10.0.5) and bootstrap tests (1000 replicates) based on the Kimura two-parameter model. Only bootstrap values greater than 60% are shown.

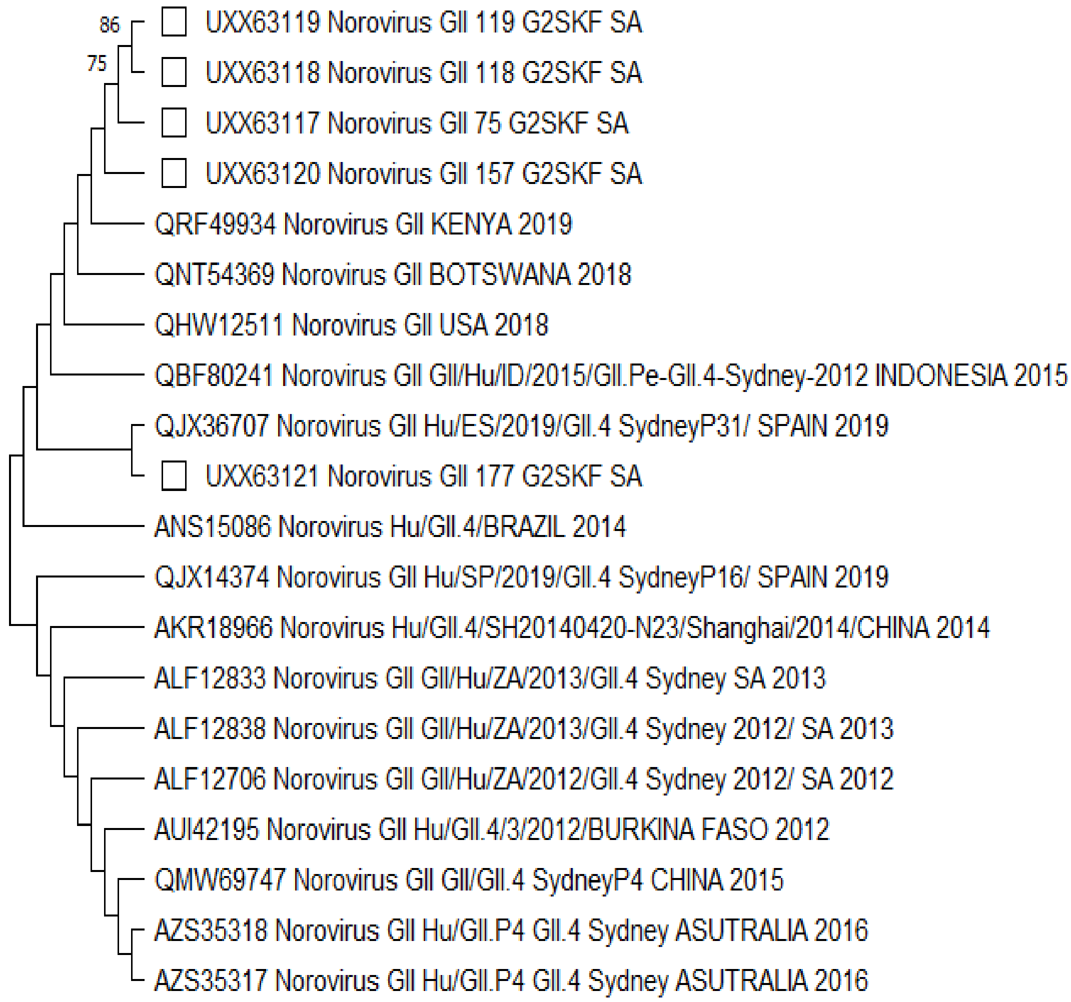

Figure S4. Phylogenetic analysis of capsid region proteins of HNoV GII.4 Sydney 2012 strains circulating in Vhembe district (South Africa) in 2019-2021. Phylogenetic tree was set using Neighbor-Joining method. Round black dots represent HNoV genotypes obtained during this investigation. Reference protein sequences were obtained from GenBank with their respective accession numbers based on nucleotide similarity with our study sequences when constructing a nucleotide based tree. All positions containing gaps and missing data were eliminated. The evolutionary distances were computed using the *p*-distance method and are in the units of the number of base differences per site. Evolutionary analyses were conducted in MEGA 11 (10.0.5) and bootstrap tests (1000 replicates) based on the Kimura two-parameter model. Only bootstrap values greater than 60% are shown.
